# Interrater Reliability of the Occupational Therapy Anticipatory Awareness Test: A Performance-Based Cognitive Assessment

**DOI:** 10.3390/brainsci15050511

**Published:** 2025-05-16

**Authors:** Danielle Mahoney, Stephanie Alvarado, Rochelle Mendonca

**Affiliations:** 1 Department of Rehabilitation and Regenerative Medicine, Programs in Occupational Therapy, Columbia University, New York, NY 10032, USA; rm3736@cumc.columbia.edu; 2 WellCare Pediatric Therapy, Colorado Springs, CO 80915, USA; steph.alvarado@yahoo.com

**Keywords:** assessment, cognition, occupational therapy, acquired brain injury, awareness

## Abstract

**Background:** Functional cognitive impairments caused by acquired brain injury (ABI) negatively impact an individual’s daily functioning. Impaired self-awareness can pose obstacles to task execution and participation. Traditional assessment methods for self-awareness lack a functional approach, highlighting the need for performance-based assessments such as the Occupational Therapy Anticipatory Awareness Test (OTAAT). The purpose of this study was to establish the interrater reliability of the OTAAT in adults with ABI. **Methods:** This was a two-phase study. Phase One involved the recruitment of three participants with ABI, who then underwent OTAAT administration within a setting of their choosing. OTAAT performances were recorded. In Phase Two, ten occupational therapists assessed the Phase One participants’ performances using the OTAAT. Phase Two’s raters’ outcomes were obtained by research personnel. These data were then collected and analyzed by research staff to measure interrater reliability using the Intraclass Correlation Coefficient (ICC) via IBM Statistical Package for the Social Sciences (SPSS) software. A two-way random effect, absolute agreement, multiple-rater/measurement ICC was adopted for this study. **Results:** Data analysis demonstrated strong interrater reliability for the OTAAT, demonstrating its consistency to measure self-awareness in individuals with ABI across different raters. Specifically, the ICC values indicated strong agreement among raters in their assessment of participants’ self-awareness performance using the OTAAT. **Conclusions:** The OTAAT has strong interrater reliability and holds promise as a valuable addition to neurorehabilitation practice. This study demonstrates the reliability of the OTAAT as a tool for assessing self-awareness in the ABI population.

## 1. Introduction

Acquired brain injury (ABI) is one of the primary contributors to both fatalities and disabilities on a global scale [1]. ABI is an umbrella term used to describe brain injuries that occur after birth and are not a result of a congenital disorder; they occur due to traumatic brain injury (TBI) or disease processes, such as a stroke, tumor, aneurysm, infection, or restriction on the oxygen being supplied to the brain [2]. According to the most recent statistics provided by the Center for Disease Control and Prevention, approximately 5.3 million Americans live with a disability secondary to an ABI [3]. Among the disabilities acquired from traumatic brain injuries, cognitive deficits are often the most debilitating [4]. Deficits in cognition significantly impact participation in several types of occupations, such as activities of daily living (ADLs), instrumental activities of daily living (IADLs), social participation, employment, and leisure participation [5,6]. These individuals often experience occupational challenges and dysfunction due to decreased functional cognition [7]. Functional cognition is defined as a person’s capacity to apply their current level of abilities and overall function to perform essential tasks across environments [8]. Self-awareness is an important component of functional cognition that is required to maintain safe and optimal participation in all daily life activities [7].

Self-awareness is defined as the capacity to recognize how impairments impact daily living [9]. When self-awareness is impaired, individuals are unable to accurately assess their capabilities and successfully adapt to changes in the environment or tasks when participating in activities of daily living. Deficits in self-awareness can lead to decreased safety awareness, difficulty with maintaining interpersonal relationships, and challenges with participation in ADLs and IADLs [7,10,11]. Lamberts et al. [12] and Giles et al. [10] highlight self-awareness to be a vital component in facilitating successful participation in these activities for patients with acquired brain injury.

Self-awareness has traditionally been viewed through a hierarchy of awareness that has been observed by practitioners within the clinical setting [13]. According to this hierarchy, individuals must first obtain intellectual awareness, which pertains to basic knowledge of existing deficits and is considered the initial level of awareness in the hierarchy. For example, if a practitioner is working in an acute care hospital and a patient can verbalize that they cannot walk to turn on the television because they have a brain injury, this would demonstrate intellectual awareness. Emergent awareness follows, this allows individuals to self-monitor their performance during a task and identify areas of challenge while it is occurring during task performance [14]. For example, while performing a dressing task, a patient states “I need help, I am unable to put my arm into the shirt because my right arm is weak”, demonstrating emergent awareness. Anticipatory awareness is an individual’s ability to predict challenges and consider task demands prior to participation, as well as anticipate possible problems in the future [14]. For example, an individual is aware that they have difficulty with their memory, so they make a list of their groceries prior to going to the store so that they can remember the items that they need; this demonstrates anticipatory awareness. Challenges with being able to recognize cognitive changes at any of these levels of self-awareness can have a significant impact on rehabilitative outcomes for individuals with ABI, making it more difficult to participate in meaningful occupations and daily routines [15].

Occupational therapists play a vital role in assessing individuals’ self-awareness during rehabilitation to further improve their participation and performance in meaningful occupations. Accurately assessing a patient’s current state of self-awareness is an important area of professional development for all occupational therapists who treat ABI survivors. Function- and performance-based assessment of self-awareness is paramount in occupational therapy practice, as it provides valuable insights into a patient’s true abilities and potential for functional independence. Unlike traditional assessment methods that rely solely on interviews, self-report questionnaires, and caregiver reports, a functional approach focuses on evaluating an individual’s performance in real-life situations and meaningful occupations. However, despite the recognized importance of functional assessment, many available assessment tools for individuals with self-awareness deficits are not occupation- or performance-based. This gap in practice highlights the need for occupational therapists to advocate for the integration of functional assessment methods into clinical practice, particularly when working with populations such as ABI survivors [16,17].

A scoping review examining self-awareness assessments for individuals with acquired brain injury [16] revealed that of the nine assessments reviewed, only one instrument, the Self-Regulation Skills Interview, assesses both emergent and anticipatory awareness [18]. The Assessment of Awareness of Deficits measures both intellectual and emergent awareness, but not anticipatory awareness [19]. All seven other assessments do not measure anticipatory awareness; they can only yield basic data about the patients’ abilities to recognize a deficit’s presence [16]. The majority of assessments reviewed relied on interview or questionnaire formats to evaluate self-awareness. Although interviews and questionnaires provide valuable information to occupational therapy practitioners, it is difficult to assess a person’s level of cognition, specifically self-awareness, from this alone [20]. Interviews and questionnaires rely largely on a person’s perspective, either the person themselves or a caregiver. Observation of the person participating in routine activities allows practitioners to gain an accurate understanding of their level of self-awareness and assess occupational areas that are impacted by deficits within this scope of cognition. Deficits in cognition have long been considered best tested through ecologically valid and occupation-based tasks due to their ability to reflect the impact of cognitive deficits on ADLs and IADLs [20,21]. This is why direct observation of daily task performance is recommended in conjunction with neuropsychological testing during the assessment of how deficits impact participation in daily routines and tasks [20].

The Occupational Therapy Anticipatory Awareness Test (OTAAT) is a performance-based assessment that provides occupational therapists with increased insight into how deficits in self-awareness impact performance and participation in functional tasks. The OTAAT utilizes functional tasks to evaluate self-awareness, while also presenting patients with tasks designed to assess their level of awareness, including anticipatory awareness. The assessment is currently intended for use with individuals aged 18 years and older who have sustained an ABI and are presenting with deficits in self-awareness [7]. Through the implementation of the OTAAT as an assessment tool, occupational therapists can gain insight into a patient’s level of self-awareness and need for support as they move forward in the rehabilitative process [7].

The OTAAT’s content validity has been evaluated in prior research [7]; however, aspects of its reliability, particularly its interrater reliability, have not been previously assessed. Interrater reliability refers to the extent of agreement or consistency among different raters or observers when assessing the same phenomenon or using the same assessment tool [22]. It is a fundamental aspect of a measure’s psychometrics, ensuring that assessments used during evaluation and their results are reliable. Interrater reliability serves as a cornerstone for establishing the consistency and accuracy of assessment outcomes, thereby guiding clinical decision-making processes, and facilitating effective communication and collaboration among multidisciplinary teams. Reliable assessment tools are essential for guiding clinical decision-making processes and optimizing patient outcomes, especially in occupational therapy, where interventions are tailored to individual needs; therefore, consistent assessment results enable practitioners to make informed decisions about treatment planning, goal setting, and intervention strategies [23].

In occupational therapy practice, where interventions are aimed at promoting participation in meaningful activities and improving quality of life, the establishment of interrater reliability for an assessment can hold particular significance. Occupational therapists rely on assessment tools to identify patients’ strengths, limitations, and areas requiring intervention. By ensuring the consistency and validity of assessment results, therapists can be confident in using the assessment to make informed decisions about intervention planning, monitor progress over time, and adjust treatment strategies as needed. The purpose of the current study is to assess the interrater reliability of the OTAAT with adults with ABI.

## 2. Materials and Methods

This study was approved by the Columbia University Institutional Review Board, protocol number AAAU4609. All subjects acknowledged their understanding of study expectations, benefits, and risks through written consent and were able to withdraw participation from the study at any time. Informed consent was obtained from all participants in both Phase One and Phase Two.

### 2.1. Phase One: Administration of the OTAAT

For the initial phase of the study, three adult participants diagnosed with acquired brain injury (ABI) were recruited to undergo the assessment (OTAAT). Inclusion criteria required that participants have a current or history of a previous ABI, be aged 18 years or older, and demonstrate a baseline level of intellectual awareness. The determination of intellectual awareness was conducted by the principal investigator, employing clinical reasoning techniques during pre-administration exercises (i.e., could the participants verbalize the existence of a brain injury and list any functional impacts from the brain injury). Participants were excluded from Phase One of the study if they did not have a current or history of an ABI, did not demonstrate intellectual awareness, and were younger than 18 years of age. Recruitment efforts for Phase One were multifaceted, incorporating email correspondence, telephone communication, and in-person engagement by the principal investigator. Prospective participants received detailed information regarding the study objectives, procedures, and their rights as research participants. Prior to enrollment, each participant underwent a pre-screening process to make sure they were a best fit for the study and met all inclusion criteria. If participants passed the pre-screening process, they were then required to provide informed consent to move further on with the study, signifying their voluntary participation in the research study.

Participants in Phase One went thorough pre-briefing on what to expect during their participation in the study. This pre-briefing and information session, facilitated by the principal investigator, encompassed detailed instruction on ethical considerations, confidentiality protocols, and parts of the assessment they would be participating in. The OTAAT was then conducted in varied settings, chosen by the participants themselves, to ensure ecological validity and relevance to real-world practice scenarios. This approach allowed participants to engage in the assessment process within contexts that were familiar and conducive to their comfort, thereby facilitating optimal performance during the evaluation. The principal investigator administered the OTAAT to the three participants, with each session recorded for subsequent analysis during Phase Two.

### 2.2. Phase Two: Scoring of Phase One Participants

In the second phase of the study, a cohort of 10 licensed and registered occupational therapists, with diverse backgrounds within the field of occupational therapy, were recruited to assess the performance of participants from Phase One using the Occupational Therapy Anticipatory Awareness Test (OTAAT) (see Table 1). Recruitment efforts were conducted through email correspondence and community outreach via fliers by research staff. Potential participants were screened against predetermined inclusion criteria. To be eligible for participation, therapists were required to demonstrate a professional interest in neurorehabilitation and possess experience in working with the adult population as practitioners. Notably, no specific practice setting was mandated, reflecting the intended versatility of the OTAAT across diverse rehabilitative environments. Individuals who passed the screening were then provided with a video session, on a secure video platform, to go over the consent form and study procedures. Procedures, risks, and benefits were reviewed with participants prior to obtaining their written consent. Consent was acquired from all participants prior to beginning participation in any part of the study and signed consent forms were sent to research staff electronically.

The participating therapists in Phase Two exhibited a range of experience in occupational therapy, spanning from 4 to 43 years. Prior to the practitioners scoring Phase One participants, they underwent training in the administration manual of the OTAAT. Subsequently, they were provided access to three videos via a secure platform compliant with the Health Insurance Portability and Accountability Act (HIPAA), enabling them to observe and evaluate the performance of Phase One individuals using OTAAT score sheets. Score sheets were then collected for data analysis. Raters were blinded to each other’s scores to reduce bias by participating in individual video sessions with research staff.

Following the completion of data collection in Phase Two, the compiled information and scores obtained from the OTAAT assessment went through rigorous analysis. The final sample for the study comprised a total of 13 participants, with three individuals from Phase One and 10 occupational therapists recruited for Phase Two.

### 2.3. Instruments

The Occupational Therapy Anticipatory Awareness Test (OTAAT) is a performance-based assessment that utilizes occupational performance as the primary context for assessing all 3 levels of awareness. This assessment tool utilizes self-rating, observation, and interview to gather information. The OTAAT is divided into two tasks: Task 1: Storing Items and Task 2: Medication Management. The “Storing Items” task requires the participant to select 4 items from a group of 10 (these items are retrieved by the therapist within the participant’s natural environment prior to the assessment beginning) and actively store these items away where they think they belong within the natural environment (i.e., their home, hospital room, etc.). This is more of a motor-focused task. While completing this task, the participant is prompted with questions such as “how easy or difficult do you think it will be completing this task?” (the participant uses a rating scale developed for the purposes of this assessment); “what areas do you think you will have difficulty with?” (the participant gives a qualitative response, and the therapist scores the response on a rating scale developed for the purposes of this assessment); “how do you think you are doing with this task?” (the participant uses a rating scale developed for the purposes of this assessment), etc. The “Medication Management” task requires the participant to organize 3 medications for the week pertaining to their prescriptions on medicine bottles. This is more of a cognition-focused task. While completing the task, the participant is prompted with the same questions listed above to assess their self-awareness. By having both motor-focused and cognition-focused tasks, the practitioner can assess if there is a difference between the patient’s self-awareness of motor deficits vs. cognitive deficits. The prompted questions are divided into three levels of awareness: intellectual, emergent, and anticipatory. For every part of the task, the therapist is required to rate the patient on their level of awareness based on observation, task performance, and clinical reasoning.

The OTAAT has been utilized with individuals over the age of 18 who demonstrate deficits in self-awareness or difficulty with recognizing changes in cognitive abilities post injury. Practitioners should apply clinical reasoning to decide if this assessment tool is appropriate for their patient. It is recommended that intellectual awareness is demonstrated by the patient to ensure the most effective use of this tool. Intellectual awareness involves recognizing, to some extent, that a function is impaired and that one is having difficulties with performance and participation in specific occupations [14]. This awareness holds significance as it serves as a foundational step; acknowledging a problem’s existence is a prerequisite for effectively anticipating potential issues during future occupational tasks.

The purpose of the OTAAT is to assess whether the patient can anticipate any difficulties or barriers that may occur prior to or while completing the task, therefore demonstrating self-awareness. It was created with the intent of being used in a variety of rehabilitative settings, as well as in natural environments. Administration of the OTAAT should take approximately 30–45 min, depending on how quickly the patient is able to perform each task and then transfer information to a scoring sheet.

### 2.4. Data Collection

Upon enrollment, the ten participating occupational therapists were provided with the necessary materials electronically by the research staff. These materials included the OTAAT Therapist Guide, the OTAAT scoring sheet, and the OTAAT administration manual. The therapists printed out these forms to facilitate the scoring process during their evaluation of the three Phase One participants. Utilizing physical copies of the OTAAT, the Phase Two participants systematically scored the performance of all three Phase One individuals based on the criteria outlined in the administration manual. The therapists then transferred these scores to the provided score sheets, ensuring accuracy and consistency in data recording. To maintain integrity and timeliness, Phase Two participants were required to submit both the completed OTAAT forms and the corresponding score sheets to the research staff within 48 h of their participation in the study. All documentation was sent to the research staff electronically.

Upon receipt of the completed materials, research staff compiled the data for subsequent analysis. Scores from the collected score sheets were transcribed into an Excel spreadsheet, where they were organized and categorized by Phase One participant number (participant 1, participant 2, and participant 3). The Excel sheets utilized for data collection and compilation did not contain any identifiable participant information, but rather employed participant numbers to maintain confidentiality and anonymity. This systematic organization and streamlined process of data collection facilitated efficient preparation for subsequent data analysis.

### 2.5. Data Analysis

Analysis was performed through the use of IBM Statistical Package for the Social Sciences (SPSS) software 29.0. The Intraclass Correlation Coefficient (ICC) was selected as the preferred statistical measure for this study due to its reliability and robustness [24]. The ICC accommodates multiple raters and the assessment of diverse sets of scores [24,25]. Given the nature of this study, a statistical method prioritizing agreement among quantitative data sharing the same measurement instrument was essential.

In selecting the appropriate ICC type for assessing interrater reliability, the authors adhered to current guidelines outlined in the current literature [25]. Factors such as the level of measurement, the purpose of the data, and the interrater reliability index were deliberated to ensure methodological rigor [25,26]. Given the ordinal nature of the data and involvement of multiple raters, a two-way random effect, absolute agreement ICC model was deemed most suitable for this study. The authors acknowledge that the sample size was small and included 10 raters, all of whom rated 3 participants, which could lead to larger random errors and imprecise estimates of variability, thus impacting the reliability of the ICC. The ICC results were interpreted in the context of the confidence intervals to determine the preciseness of the results.

## 3. Results

Analysis of the study data unveiled significant consensus among raters, confirming the strong interrater reliability of the OTAAT (see Table 2). Notably, the lowest average measure ICC observed among participants was 0.960 (CI: 0.914–0.986), which indicates excellent interrater reliability. Moreover, the data demonstrated an average Intraclass Correlation Coefficient (ICC) of 0.975, indicating excellent interrater reliability for the OTAAT and underscoring its intent at evaluating self-awareness in individuals with ABI. The confidence intervals for all three participants across the 10 raters were narrow, and all were within the range of excellent reliability, indicating that the ICC estimates were precise, despite the small sample size.

## 4. Discussion

The objective of this study was to evaluate the interrater reliability of the OTAAT. The OTAAT exhibits strong interrater reliability, indicating its value in neurorehabilitation assessment. Raters exhibited the highest agreement on questions related to “Participant Response”, where participants rated their own performance using a visual scale from 1 to 5. This indicates the scale’s clarity and usability. Additionally, strong agreement was observed during steps 3 and 8 of the OTAAT, which assessed anticipatory awareness during specific tasks. Notably, higher agreement was noted during step 8, which involved the “Medication Management” task. Moderate agreement was observed among raters for therapist response questions immediately following participant responses (e.g., 5B and 10B). These questions required therapists to assess the patient’s emergent awareness after the participant rated their awareness on the current task. 

Overall, ICC average measures ranged from 0.960 to 0.993, indicating excellent interrater reliability across participants and raters. Participant three in Phase One had the lowest ICC value, suggesting less agreement among raters during scoring. Potential reasons for this disparity include differences in rater experience or clarity regarding participant three’s performance and responses during OTAAT administration. 

The levels of agreement observed in the study can be attributed to several factors. Firstly, the structured nature of the OTAAT, with clear guidelines and scoring criteria, likely contributed to consistent scores among raters. Additionally, the use of visual scales and specific task prompts may have facilitated clearer understanding and interpretation of participant responses, enhancing agreement among raters.

To improve the reliability of this outcome measure further, several strategies can be considered. Refining the OTAAT manual to clarify ambiguous instructions or explicit guidelines on patient prompting, may improve the use of the OTAAT among clinicians. This could involve simplifying language, clarifying scoring criteria, providing examples for complex scenarios, or offering additional guidance on how to interpret participant responses in certain contexts. Moreover, ongoing supervision and support from experienced clinicians within the area of self-regulation can provide valuable guidance to practitioners, particularly those with limited experience. Encouraging open communication and feedback among occupational therapists working within the field of cognition and self-awareness can also foster a collaborative environment where they feel comfortable discussing challenges and seeking clarification when needed.

Raters requested a final score regarding the patient’s overall awareness during the OTAAT, but the assessment was not designed to provide such an outcome. The OTAAT serves as a tool to assess the patient’s awareness level in relation to their performance. The authors plan to collaborate with clinicians to determine what type of summary statement(s) would be useful regarding a patient’s self-awareness for documentation, reimbursement, and summarizing purposes.

### Limitations

This study was not without its limitations. Raters expressed uncertainty about when to provide cues to participants and introduce compensatory strategies outlined in the manual, such as the “Tool Box”. To address this issue and enhance scoring reliability, the manual will be revised to provide clearer guidance on when to cue participants and when it is appropriate to introduce or utilize the compensatory strategies.

Furthermore, this study’s limitations extend to the varying levels of experience among Phase Two participants in the field of neurorehabilitation. Occupational therapy experience among participants ranged from 3 to 45 years. Differences in occupational therapy experience can influence practitioners’ judgments of self-awareness due to variations in their understanding of this cognitive domain. As a result, these differences in experience may lead to discrepancies in scoring. 

Conducting sessions for Phase Two raters over Zoom rather than in person could have posed several limitations. The limitations of conducting Phase Two participants’ sessions over Zoom left room for confusion regarding what Phase One participants said during videos, depending on Phase Two raters’ sound quality and the device they used during the sessions. This could have resulted in inconsistencies in the information conveyed to Phase Two participants. Additionally, the lack of face-to-face interaction during this phase of the study could have impeded participants’ ability to ask questions or seek clarification in real time from the research team, limiting their engagement and understanding of the OTAAT procedures.

Lastly, the sample size was small with 3 participants and 10 raters, and therefore, the results should be interpreted cautiously. Even though the narrow width and range of the confidence intervals indicate preciseness in ICC estimates, the authors acknowledge that this study was a pilot study and requires larger samples in the future.

## 5. Conclusions

This study showcases the reliability of the OTAAT as a performance-based tool for assessing various levels of self-awareness in individuals with ABI. The significant interrater reliability observed underscores the versatility of this assessment tool across different settings and among practitioners with diverse levels of experience and specialties in neurorehabilitation. By providing functional and valid assessment options, the OTAAT has the capacity to enhance accessibility to effective tools for occupational therapists in the realm of self-awareness assessment.

## Figures and Tables

**Table 1:** Phase Two participant demographics.

Participant Number	Years of Experience	Area of Practice within Cognition
1	4	Acute Care Rehabilitation
2	4	Home Health Rehabilitation
3	22	Inpatient Rehabilitation
4	12	Inpatient Rehabilitation
5	43	Private Practice
6	3.5	Day Rehabilitation
7	19	Academia
8	5	Inpatient Rehabilitation
9	10	Acute Care Rehabilitation
10	8	Academia

**Table 2:** Data analysis results.

Phase One Participant	ICC Average Measure	95% Confidence Interval
1	0.971 *	0.938–0.990
2	0.993 *	0.985–0.998
3	0.960 *	0.914–0.986

* <0.001 significance.

## Data Availability

The original contributions presented in this study are included in the article. Further inquiries can be directed to the corresponding author.
